# Physical Literacy and Obesity Risk in Children: A Systematic Review

**DOI:** 10.3390/ijerph23050562

**Published:** 2026-04-27

**Authors:** Lauren Callaghan, Anushree Dwivedi, Cathal Óg O’Sullivan, Saim Ghafoor, Michelle Queally, Robert Mooney

**Affiliations:** 1 Department of Sport, Exercise & Nutrition, Atlantic Technological University, H91 T8NW Galway, Ireland; cathalog.osullivan@atu.ie (C.Ó.O.); robert.mooney@atu.ie (R.M.); 2 Department of Mechanical and Industrial Engineering, Atlantic Technological University, H91 T8NW Galway, Ireland; anushree.dwivedi@atu.ie; 3 Department of Computing, Atlantic Technological University, F92 FC93 Donegal, Ireland; saim.ghafoor@atu.ie; 4 Department of Enterprise and Technology, Atlantic Technological University, H91 T8NW Galway, Ireland; michelle.queally@atu.ie

**Keywords:** physical literacy, childhood obesity, physical activity, assessment tools

## Abstract

**Highlights:**

**Public health relevance—How does this work relate to a public health issue?**

- This systematic review examines the link between physical literacy and childhood obesity risk amid rising overweight and declining physical activity.
- It evaluates physical literacy assessment tools measuring competence, confidence, motivation, and engagement to inform population-level interventions.

**Public health significance—Why is this work of significance to public health?**

- Physical literacy represents a modifiable factor influencing children’s physical activity and long-term health behaviours.
- Assessing current tools supports accurate measurement and the development of effective strategies to prevent obesity.

**Public health implications—What are the key implications or messages for practitioners, policy makers and/or researchers in public health?**

- Enhancing physical literacy in childhood may promote sustained physical activity and reduce obesity risk across the life course.
- Identifying robust assessment tools is essential for monitoring development, evaluating interventions and informing evidence-based policy and practice.

**Abstract:**

(1) Background: Childhood obesity is one of the greatest challenges facing public health in the 21st century. Currently, there is a great deal of interest in the concept of physical literacy due to its comprehensive nature on the development of physical activity. Physical literacy can be described as the motivation, confidence, physical competence, knowledge and understanding to value and take responsibility for engagement in physical activities for life. The aim of this paper is to provide a systematic review of the literature examining the relationship between physical literacy and risk of obesity in children. This paper also focuses on providing an evaluation of the availability and effectiveness of current physical literacy assessment tools. (2) Methods: This review was conducted in accordance with the PRISMA (Preferred Reporting Items for Systematic Reviews and Meta-Analyses) guidelines. (3) Results: A total of 3267 papers were identified from five major databases. Twelve studies met the inclusion criteria, with nine studies included in the final review following quality appraisal. (4) Conclusions: This review identified significant gaps in our understanding of physical literacy emphasising the need for a consistent framework, standardised assessment tools and more experimental research.

## 1. Introduction

Childhood obesity is a major public health issue of the 21st century with chronic adverse consequences [1]. The World Health Organisation (WHO) defines being overweight and obese as an abnormal or excessive accumulation of body fat that poses health risks [2]. The latest data from the WHO indicates that 390 million children and adolescents are classified as overweight, including 160 million living with obesity [3]. This number is projected to increase to 254 million by 2030 [4]. These alarming trends provide a clear need for solutions to avoid catastrophic societal issues for future generations.

Although childhood obesity is a complex and multifactorial disease, its development is primarily driven by an imbalance between energy intake and energy expenditure [5]. Physical activity (PA) constitutes 25% of total energy expenditure [6] and is essential for the normal growth and development of children [7,8,9,10]. However, despite widespread awareness and research, physical inactivity during childhood continues to be a growing health issue presenting significant challenges [11]. Globally, over 80% of children and adolescents are classified as physically inactive [12] with activity levels declining significantly around age six and again at age 13, particularly among girls [13,14,15]. Longitudinal research has indicated that children with obesity are significantly more likely to remain obese into adulthood [16]. More concerningly, findings indicate that individuals who were obese during childhood face elevated risks of morbidity and mortality, regardless of their weight status during adulthood [17].

**Figure 1:**
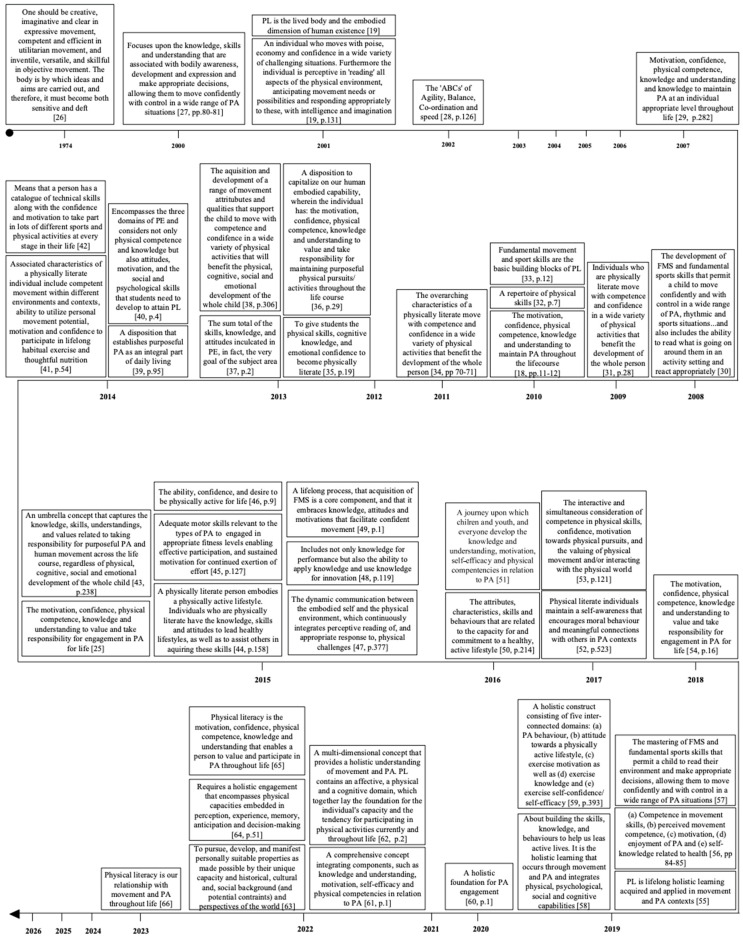
The evolution of the physical literacy definition. References are presented in chronological order (PL; physical literacy, PA; physical activity; FMS: fundamental movement skill) [18,19,25,26,27,28,29,30,31,32,33,34,35,36,37,38,39,40,41,42,43,44,45,46,47,48,49,50,51,52,53,54,55,56,57,58,59,60,61,62,63,64,65,66].

The term physical literacy (PL) has gained prominence as a concept to aid the understanding of children’s engagement, or lack thereof, in PA [18]. Arguably the most seminal contribution to this concept is the work of Margaret Whitehead who conceptualised the initial philosophical definition of PL and has subsequently refined it in recent years. Physical literacy was defined as the lived body and the embodied dimension of human existence [19]. Although, this philosophical perspective laid the foundation for understanding PL as a holistic phenomenon, it offered limited guidance for practitioners and created a gap between theoretical intent and practical application. Consequently, the revision of the PL definition has become the subject of considerable scholarly debate, resulting in a multitude of definitions and interpretations (Figure 1). A total of 45 definitions have been proposed in recent decades, reflecting the variation in philosophical perspectives by numerous research groups and country-specific conceptualisations. With no consistent agreement regarding the PL concept or definition, it makes it very difficult, if not impossible, to agree on a common framework or approach to finding solutions.

Recent definitions of PL do largely agree on a core set of components. Physical literacy is portrayed as a holistic construct that emphasises physical, cognitive and affective domains that support engagement is PA throughout the life course [20,21,22]. However, differences between definitions are evident in the inclusion of the social domain. This domain is inaccurately represented, being acknowledged in some frameworks but absent in others [23,24]. This raises several implications. Namely, the inconsistent inclusion of this domain contributes to the ambiguity within PL, making it difficult to develop a universally accepted definition. This review adopts the definition of the International Physical Literacy Association (IPLA) which defines PL as the “motivation, confidence, physical competence, knowledge, and understanding to value and take responsibility for engagement in physical activities for life” [25] (p.1). Arguably, this conceptualisation provides a more comprehensive and multidimensional lens of PL, going beyond that of physical and motor skills to include meaningful engagement with PA across an individual’s lifespan.

In addition to problems with the definition, there are also complications with existing tools measuring PL. Although, it is encouraging that a number of assessment tools have been developed over the past decade, none are without limitations and no common approach exists. The Canadian Assessment of Physical Literacy (CAPL) [67], the International Physical Literacy Association (IPLA) Physical Literacy Matrix [68] and the passport for Life [51] were identified as the only tools that assess the three domains of PL as a whole [69]. Whilst other tools exist, they assess one or two domains related to PL while marginally assessing the others, contradicting the multidimensional concept of PL. For example, the Fundamental Movement Skills: 60 Minute Kids’ Club (60 MKC) [70], conceptual model of observed physical literacy (CMOPL) [44], and physical literacy observation tool (PLOT) [71] assesses the physical competence component of PL, whereas the Physical Literacy Assessment for Youth (PLAY) [72], the Perceived Physical Literacy Inventory (PPLI) [73], national standards for K-12 physical education and PE metrics [74] and the Chinese Assessment and Evaluation of Physical Literacy (CAEPL) [20] assess a combination of physical competence and cognitive domains.

Despite PL being recommended for all ages, childhood has been identified as a critical phase for the development of attributes that contribute to lifelong PA [75,76]. Findings suggest that individuals with high levels of PL are more likely to meet recommended PA levels [77]. Therefore, early-age PL development could be an effective strategy to increase levels of PA. This is important as regular PA is widely recognised as an important component of healthy weight management and obesity prevention [78]. As such, this may benefit the health of children, especially those with a high risk profile (high adiposity, low fitness, and low PA levels) in an attempt to reduce or stabilise the current obesity trajectory in children. If early PL development leads to PA improvements and long-term health outcomes, it could justify PL being positioned as a public health target, warranting integration into school curricula, which could lead to increased community health initiatives. This may contribute to lowering the current economic and health burden of obesity and promote health equity through lifelong PA habits beginning in childhood. There remains a notable gap within this area of research, namely a systematic review that critically explores the relationship between PL and risk of obesity in children. This absence renders it difficult for researchers to form a comprehensive understanding of the overall relationship, if any, as current findings remain scattered, thereby limiting the development of effective interventions. The aim of this review is to systematically and critically examine the existing literature on PL and its association with childhood obesity, addressing the gap in existing knowledge, and to provide a deeper understanding informing the development of future interventions.

## 2. Materials and Methods

### 2.1. Research Questions

A systematic review of the literature was conducted in an attempt to answer the following three research questions: (1) What is the availability of current PL assessment tools and how are they characterised? (2) What is the effectiveness of current PL assessment tools? and (3) Is there a relationship between PL and the risk of obesity in children?

### 2.2. Study Design

The design and reporting of this review followed the PRISMA (Preferred Reporting Items for Systematic Reviews and Meta-Analyses) guidelines [79]; the completed checklist is provided in Supplementary Table S2. The article selection involved three stages: (i) an initial screening of article titles across multiple databases, with results merged and duplicates excluded; (ii) a review of the titles and abstracts of the selected articles; and (iii) a full-text review of the selected articles based on predefined inclusion and exclusion criteria.

### 2.3. Search Strategy

A comprehensive search was conducted on the following databases: (1) PubMed; (2) ScienceDirect; (3) Scopus; (4) Web of Science and (5) Sport Discus. These databases report on areas including education, sports and health, which are all relevant to the concept of PL and childhood obesity and therefore increased the likelihood that all relevant studies were located. The keyword string used for the search was “(childhood obesity OR paediatric obesity OR juvenile obesity OR youth obesity) AND (physical literacy OR movement control OR functional movement) AND (relationship OR connection OR association OR link)”.

### 2.4. Inclusion and Exclusion Criteria

Studies were included if they focused on children aged five to 13 years who were either classified as having obesity, at risk of obesity, or experiencing weight-related concerns. Studies including broader age ranges were also eligible where the sample over-lapped with the predefined age range or were age-related analyses were reported. Eligible studies reported outcomes related to childhood obesity such as body mass index (BMI), body fat percentage or other comparable measures. Studies examining the influence of PL on PA levels, sedentary behaviour or related health outcomes were considered for inclusion. All publications from January 2012 to November 2025 were included in the search. Exclusion criteria were applied to studies involving populations older than 13 years or children with physical disabilities not associated with obesity. Studies focusing exclusively on other contributing factors such as nutrition, psychological influences or physical activity without integrating PL were also excluded. Filters were applied to limit search results to English-language publications in academic journals. A random sample of the initially selected studies (*n* = 50) was independently reviewed by two members of the research team as part of quality control procedures. Inter-rater agreement was 100%, with no discrepancies identified, and all studies were confirmed to meet the predefined inclusion criteria.

### 2.5. Quantitative Synthesis

A quantitative synthesis was considered by the authors when conducting this review. However, substantial heterogeneity was identified across the included studies. Variations in study design, anthropometric measures and measurement tools used to assess PL limited the comparability of outcomes. Furthermore, there was a lack of sufficient statistical data to support pooling of results. Given these factors, conducting a meta-analysis was deemed inappropriate, as it may have produced misleading conclusions. Therefore, a narrative synthesis was deemed more appropriate.

## 3. Results

**Figure 2:**
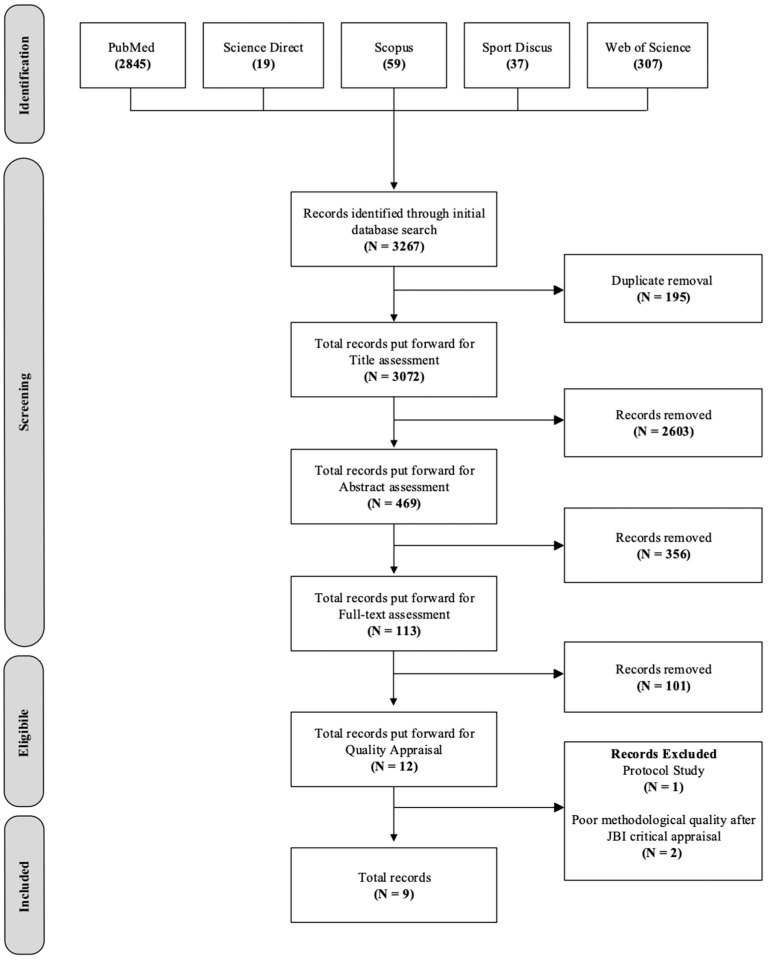
Systematic review search strategy and results.

A process flowchart detailing the results of the database search and article selection is provided in Figure 2. The initial database search identified 3267 articles. After removing 195 duplicates, 3072 articles remained for screening. Titles were assessed against the inclusion and exclusion criteria, resulting in 469 articles progressing to abstract review. After a thorough review of full texts, the final number of publications included in this review was 12. Quality appraisal using the Joanna Briggs Institute (JBI) critical appraisal tool led to the exclusion of two studies [80,81]. The quality appraisal scores for each study are available in Supplementary Table S1. A further study was excluded as it was a protocol study, resulting in nine papers being eligible for inclusion [82]. Table 1 provides a summary of the publications selected. 

## 4. Discussion

The aim of this systematic literature review was to gather, analyse and assess the existing evidence as presented in the current literature for answering the following three research questions; (1) What is the availability of current PL assessment tools and how are they characterised? (2) What is the effectiveness of current PL assessment tools? and (3) Is there a relationship between PL and the risk of obesity in children?

### 4.1. The Availability of Physical Literacy Assessment Tools

Whilst there exists a number of assessment tools to measure PL, only four tools were featured in the reviewed studies (Table 1). This could be attributed to the strict inclusion criteria or due to the fact that the design of the featured tools aligns with the age cohort included in this paper. To the best of the authors’ knowledge, all relevant and eligible studies have been included in this review. Although several tools do not feature in this review, their omission is likely due to limitations in their design rather than a gap in the systematic search strategy. With that said, most authors selected either the Canadian Assessment of Physical Literacy (CAPL) first edition or [84] the second edition (CAPL-2) [86,87,88,90,91], whilst others opted for the Physical Literacy Assessment for Youth (PLAY) [83,85] or the French Perceived Physical Literacy Inventory (F-PPLI) [89]. The CAPL and CAPL-2 frameworks are structured around four domains, whilst both the PLAY tool and the F-PPLI incorporate three components.

**Figure 3:**
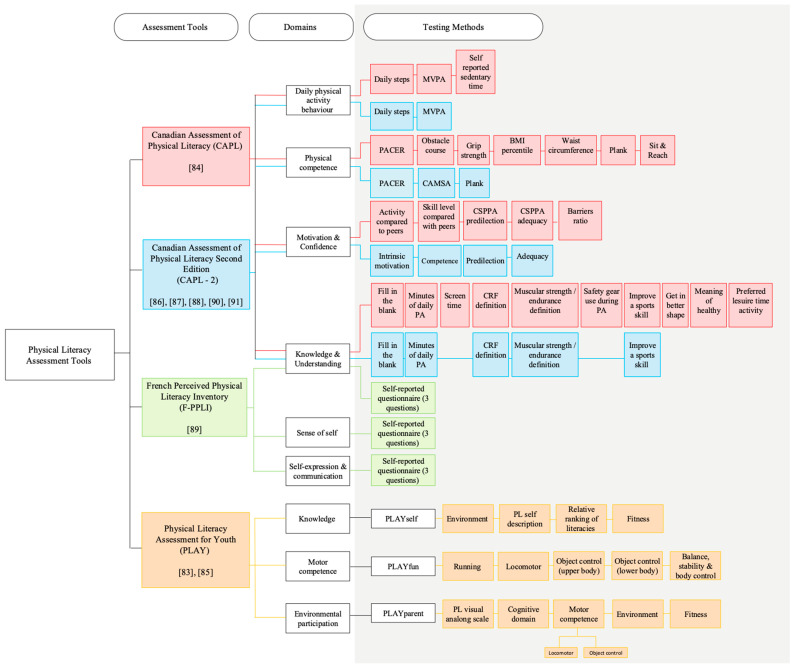
Summary of the PL assessment tools included in the selected studies, detailing the assessment domain included in each tool and tests adopted to measure each domain (PACER: Progressive Aerobic Cardiovascular Endurance Run; CAMSA: Canadian Agility and Movement Skills Assessment). The boxes are colour-coded according to assessment tool (CAPL: Red; CAPL-2: Blue; F-PPLI: Green and PLAY: Orange) [83,84,85,86,87,88,89,90,91].

An overview of each assessment tool is presented in Figure 3. There exist few commonalities among these tools. For example, the CAPL, CAPL-2 and F-PPLI include a knowledge and understanding domain, whereas the PLAY tool opted only to assess the knowledge of an individual, disregarding the broader understanding component. Similarly, the CAPL and CAPL-2 place emphasis on physical competence, whilst motor competence is assessed by PLAY tools. Moreover, differences also exist in the grounding definition underpinning each assessment tool. While the CAPL was designed to assess the multidimensional construct of PL, the reviewed tools do not all align equally with the IPLA definition and instead capture different combinations of its domains. This makes direct comparison difficult and highlights an important challenge in the field. Differences are further reflected in the specific tests used to assess each domain. Therefore, this review compares assessment tools according to their inclusion of objective and subjective measures of PL.

#### 4.1.1. Objective Measures of Assessing Physical Literacy

The CAPL assesses the three domains of PL as a whole, with individuals scored out of 100. Originally, this tool was created to address the lack of objective PL data and provides a robust and comprehensive aggregate assessment of PL [77,92,93]. Yet, contradicting this, the physical competence domain is only one of four that is solely objective, whereas the daily PA domain is partially objective. Longmuir and colleagues [94] identified several limitations with this initial model; for example, the CAPL manual instructed that the barrier instrument should be reverse-scored. This meant that the CAPL website scored the instrument correctly whilst the instructions in the manual were incorrect for those wishing to complete the scoring manually. Secondly, individuals were questioned about their preferred leisure activity, which should have assessed affective domain; however it had been placed within the cognitive domain [50].

A notable refinement in the CAPL second edition (referred to as the CAPL-2) is the modification of the daily physical activity behaviour domain that excludes the sedentary time component (Figure 3) and includes a re-weighting of the daily steps from 30 points to 32. Given that researchers have continually identified pedometers as problematic when used with a young cohort, this reweighting is concerning. Some researchers [95] have claimed that children often view these devices as toys, which leads to them being potentially disassembled or damaged, while others have critiqued their inability to detect non-locomotor movements such as swimming or cycling [96]. With that said, they may have potential in informing certain aspects of PL assessment within controlled environments. However, assigning them one-quarter of the overall assessment may be a disproportionate allocation. Caldwell and colleagues [85] expressed similar concerns, highlighting that the absence of waterproof accelerometers limits the detection of aquatic activities, leading to a potential underestimation of participant PA levels.

**Figure 4:**
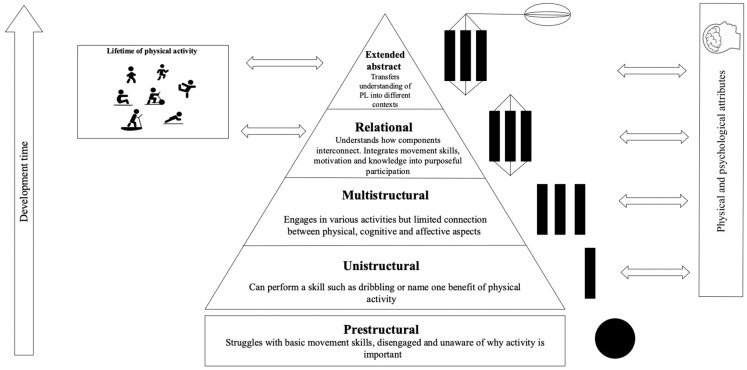
Application of the SOLO taxonomy within a PL framework. The left arrow shows progression over time and the pyramid levels reflect the stages of development that lead to lifelong engagement in PA. The horizontal double-headed arrows indicate a reciprocal relationships between the SOLO levels and associated physical and psychological attributes.

More recently, the physical domain of the CAPL-2 has come under debate, with some researchers [69] suggesting disproportionate emphasis is put on the physical component of PL. Arguably, this would contradict the holistic framework of this concept given that PL is multidimensional. Recently, Dudley and Cairney [97] have scrutinised the fact that two-thirds of the physical competence domain measures cardiovascular and muscular endurance with scores being derived from norm-referenced fitness tests. Rather, the same authors suggested that measurements be conducted in terms of learning and progression. This supports the use of a developmental framework such as the Structure of Observed Learning Outcomes (SOLO) taxonomy. This would ensure interventions move beyond that of a physical focus but also nurture the cognitive, affective and behavioural domains of PL. Figure 4 has been created as part of this review to provide a structured, progressive and holistic framework, whereby domains are not assessed in isolation but interpreted collectively across levels of progression. This would enable practitioners and researchers to assess an individual’s PL journey from basic movement awareness (i.e., prestructural) to acquiring a deep understanding of PA and associated health benefits (i.e., extended abstract).

Caldwell and colleagues [85] generated a PL composite score using the Physical Literacy Assessment for Youth (PLAY), which gains a multi-assessment perspective on PL. Objectively, 18 movement skills across five domains: running, locomotor, upper-body object control, lower-body object control and balance, stability and body control. These domains assess movement skills that are transferable across different activities and contexts, rather than being specific to one sport. However, differences among scoring protocols of this tool and both editions of the CAPL accelerate the need for a standardised tool. For example, the PLAY model scores individuals within four categories: (i) developing, if a performer exhibits numerous major gaps during execution; (ii) acquired, if there is a basic level of execution with minor sequencing errors; (iii) emerging, if there are a limited number of major gaps but the performer is able to execute basic sequencing of the task; and (iv) proficient, awarded when high proficiency is displayed. Some have argued that this tool disproportionally focuses on physical competency [39,53], whilst others [69] have critiqued it for its over-emphasis on fundamental movement skills (FMSs). This is an important limitation, as strong performance on selected physical tasks does not necessarily indicate stronger PL overall, particularly when the broader construct also includes affective, cognitive, and behavioural components. Furthermore, Lundvall and colleagues [98] have warned that FMSs should not be confused or used as a synonym for PL, while Pots et al. [99] have argued that an over-emphasis on FMSs can undermine motivation and confidence, ultimately hindering the development of PL itself. Consequently, it could become questionable if PLAY tools measure PL or aspects of PL and FMSs.

#### 4.1.2. Subjective Measures of Assessing Physical Literacy

Self-reporting is widely used as a method of measuring children’s PA due to its convenience of administration, low cost, and ability to collect a variety of PA variables over time [100]. Three tools included in this review partially depend on subjective measures, whereas the F-PPLI measures PL solely through a subjective lens. Nezondet et al. [89] used a five-point Likert scale to assess three components: (i) sense of self and self-confidence, (ii) knowledge and understanding and (iii) self-expression and communication.

Several methodological limitations were acknowledged by the authors. Most importantly, measuring moderate-to-vigorous physical activity (MVPA) through perceived and self-reported methods may lead to variations or inaccuracies in results as participants may struggle to assess their health behaviour. This is in line with findings of Sallis and colleagues [100] who reported that children are ill-equipped to accurately report the duration of PA, with some unable to tell the time or estimate it. The same authors also found that children were unable to report both the frequency and intensity of PA. This is concerning, given that both the CAPL and CAPL-2 award three points and five points, respectively, for children reporting the number of days they have engaged in MVPA for at least 60 min. This differs slightly from the multi-perspective of a child’s PL generated using PLAY tools with PLAYparent. This provides researchers with an additional perspective by identifying positive and negative factors that affect the child’s ability to lead a healthy lifestyle.

Collectively, the arguments presented highlight that the assessment of PL remains an area for ongoing research. It is evident that no gold-standard assessment tool currently exists for evaluating PL. This raises several implications for practitioners when selecting an appropriate tool. Practitioners should consider factors such as population, setting and feasibility when choosing an appropriate tool. For example, the CAPL appears an authentic assessment tool to measure the multidimensional component of PL. However, its suitability for school environments may be limited due to time demands and the use of pedometers, which may be problematic in younger populations and may lead to underestimation of daily steps if devices are forgotten. Conversely, PLAY tools offer a flexible and practical alternative, particularly in school and community settings. Although critiqued for an over-emphasis on FMSs, the inclusion of PLAYparent provides a unique perspective into a child’s daily behaviour. This multi-perspective approach makes PLAY tools especially useful in school-based contexts or community programmes when capturing broader insights into children’s movement behaviours is important. Finally, the F-PPLI may be appropriate in settings where time and resources are limited; however its subjectivity must be taken into consideration. Collectively, inconsistencies in grounding definitions, domain inclusion and assessment approaches highlight an important challenge within the field and reinforce the need for a more standardised assessment tool. This is essential for improving the accuracy of PL assessment and would support a more meaningful comparison across studies. Given the age we currently live in, technological approaches such as wearable technology may also warrant exploration in future research.

### 4.2. The Effectiveness of Physical Literacy Assessment Tools

Given the increased popularity of PL in both research and practise, academics have begun to investigate the psychometric properties of available assessment tools (Table 2 summarises the validity and reliability of the aforementioned tools). Validity was determined through the following: (a) construct; (b) convergence; and (c) content. Caldwell and colleagues [101] examined the measurement properties of PLAY tools, whereas some authors assessed the individual workbooks such as PLAYself [102] and PLAYfun [103]. Construct validity was found to be supported by positive correlations between PLAYbasic, PLAYfun and age (r = 0.16–0.32) [101]. This indicates that motor competence develops as children get older. In terms of convergent validity, positive correlations were found between each of the PLAY workbooks. Notably, PLAYself showed a significant positive correlation with PLAYfun (r = 0.52, *p* < 0.001), indicating that self-perceived PL is aligned with achieved PL [102]. This finding supports the validity of using self-report tools like PLAYself, as it demonstrates a positive association between individuals’ perceptions and their actual abilities. In addition, the validity of the CAPL-2 is well documented, with validity evidence being published for the physical literacy knowledge questionnaire (PLKQ) [104] and the overall assessment [105]. The PLKQ offered support for content validity, as knowledge was found to increase with age. Although no significance was found for movement competence, it could be argued that the PLKQ does not represent the multidimensional nature of PL, which includes an affective and physical domain. Sum et al. [73] provided validity evidence for the PPLI, finding it to be a valid measure of PL among its designated population. The authors reported moderate convergent validity supported by average variance extracted (AVE) values (0.43–0.54); although they are low, the authors found them to be a valid measure of perceived PL.

Researchers have variously used (a) inter-rater reliability; (b) internal consistency; (c) test–retest reliability and (d) item factor correlations to determine if assessment tools are reliable measures of PL. PLAYfun [101] demonstrated a strong internal structure suggesting its potential as a dependable instrument for accurately measuring PL in children. However, moderate reliability and lower internal consistency of PLAYbasic may raise concerns about its measurement precision. Partial evidence was reported for the internal structure for the PPLI [73] but it lacked reliability data. As a consequence, this may affect the tool’s overall credibility among different populations, which might make practitioners cautious about adopting this assessment tool. Moderate-to-strong test–retest reliability was reported for the PLKQ component of the CAPL [104], whilst other researchers have reported high reliability for the motivation and confidence domain (*α* = 0.90) [106].

### 4.3. Relationship Between Physical Literacy and Risk of Obesity in Children

Much of the current literature indicates a relationship between PL and body composition [85,86,89,91]. Children with low levels of PL demonstrate low physical competence, motivation and confidence, which leads to unhealthy behaviours and a lack of engagement in PA. This in turn can bolster the obesity barrier. Nevertheless, it remains premature to infer a direct relationship, as the evidence is largely based on cross-sectional work, and there is limited experimental or interventional work. As such, the current findings should be interpreted as associations between PL and the risk of obesity at the time of measurement rather than as evidence of causality.

Across the reviewed studies, moderate correlations have been reported between PL and various markers of adiposity, for example, body mass index (BMI). This was the most frequently used anthropometric measure among researchers [83,84,85,86,87,88,89,90,91] for assessing obesity, likely due to its simplicity, cost-effectiveness, and suitability for large-scale studies [107]. Whilst findings suggest that BMI is associated with lower PL scores, it is not strong enough to be considered a major determinant of PL. For instance, Liu and colleagues [87] reported weak correlations between BMI and total PL scores, as well as domain-specific scores, with the exception of the knowledge and understanding domain, which demonstrated a very weak positive correlation with BMI (r ranging from −0.171 to −0.217, with the exception of r = 0.006 for the knowledge and understanding domain). Mendoza et al. [86] reported a moderate negative association between BMI and total PL score (r = −0.466, *p* < 0.012). Again, this implies a slightly stronger association, but it reiterates that BMI is not a sufficient standalone indicator of PL levels, given that the strength of this association varies across different contexts.

As a standalone metric, BMI is an inadequate indicator for accurately classifying a child’s weight status due to its inability to distinguish between fat mass and lean mass [108]. Misclassifications can arise due to this limitation; for instance, children with high muscle mass may be categorised as overweight while those with low muscle mass but high adiposity may be misclassified as a healthy weight. Given these inconsistencies, researchers have adopted a multi-assessment approach, incorporating additional anthropometric measures to improve accuracy in assessing body composition. However, although these additional indicators may provide a more comprehensive assessment of adiposity, variability in measurement techniques may limit comparability among studies. Despite this, cardiorespiratory fitness (CRF) was the most adopted alternative to BMI [83,85,87,88,89,90], with other measures such as percentage of body fat (%BF) [83,85,88], fat mass (FM) [86], fat-free mass (FFM) [85,86], percentage of skeletal muscle mass (%SMM) [88,89] and waist circumference [83,84,87] also being used.

The relationships between PL, FM, and %FM exhibited weak-to-moderate correlations, similar to those reported for BMI. Mendoza and colleagues [86] reported that higher FM and %FM were associated with lower PL total scores (r = −0.478, *p* < 0.012 and r = −0.491, *p* < 0.012, respectively) and every domain score. For example, a moderate negative correlation for FM and %FM and the physical competence domain was reported (r = −0.471, *p* < 0.012 and r = −0.507, *p* < 0.012, respectively), indicating that children with higher FM are more likely to exhibit lower physical competence. On the other hand, a weak negative correlation between FM, %FM and the knowledge and understanding domain (r = −0.139 and r = −0.152, respectively) was reported. This lack of significant association between FM, %FM and this domain suggest that cognitive aspects of PL may develop independently of weight status. It is conceivable therefore that children with a higher body composition may still possess a similar level of theoretical knowledge about PA and health as their normal-weight peers. This is supported by Munoz-Uturbia and colleagues [91] who reported that knowledge related to PA increased with age but was not significantly associated with BMI. These findings highlight that knowledge alone may not translate into healthier weight status and potentially highlight age as a potential moderator. However, it should be noted that this study applied the CAPL-2 to an older population than designed for, which limits its comparability with other studies.

The hypothesised pathway liking PL to childhood obesity may suggest that improvements in PL enhance motivation, confidence and competence, leading to increased engagement in PA and subsequent reductions in adiposity. However, this pathway is largely theoretical within the current evidence base. The inclusion of the intervention study by Nezondet et al. [88] is therefore particularly important, as it provides preliminary evidence to support this pathway. The authors reported improvements in PL alongside reductions in anthropometric indicators. However, given that this is the only intervention study identified and that it employed a single-arm, non-randomised design, it remains premature to draw definitive conclusions. Therefore, establishing this relationship is of vital importance for the design of public health interventions and school-based programmes. If children with higher PL present healthier anthropometric measures, then early PL development could serve as a potential strategy to reduce childhood obesity levels. This would have several implications for physical education curriculum design, school-based interventions, and physical activity programmes. It is important to acknowledge that younger children have limited autonomy and control over their behaviours, with parents, guardians and the surrounding environment remaining key determinants of diet, opportunities for PA, and other related factors. Parental engagement is therefore a key factor in the prevention and management of childhood obesity, as parents substantially influence related outcomes [109]. This should be taken into account when designing interventions aimed at this population. However, if improving PL does not favour improvements in body composition, interventions based on this may be ineffective and poorly targeted, resulting in a waste of current resources.

Consequently, there remain several areas for future research. Researchers should prioritise longitudinal study designs, experimental studies, or well-designed randomised controlled trials with sufficient sample sizes to detect meaningful changes in both PL and adiposity outcomes. Studies should aim to recruit larger, diverse cohorts across school and community settings to improve generalisability. Follow-up periods of at least 6–12 months are recommended to assess short-term changes, with longer-term follow-up (≥12 months) needed to determine whether improvements in PL translate into sustained behavioural changes and reductions in adiposity over time. Furthermore, future studies should explore potential moderators, including sex, age and socioeconomic status, as these factors may influence the relationship between PL and the risk of obesity. Although one study [91] reported sex-based differences, these differences were inconsistently examined across the remaining studies. Similarly, current studies exhibit an inconsistency in the reporting of anthropometric measures, with some studies examining correlations between PL and specific obesity indicators, while others categorise participants by weight status (e.g., healthy weight or obese). While both approaches give valuable insights, this variation makes it challenging to draw definitive conclusions and develop targeted interventions within this area. Lastly, future studies should also aim to report socioeconomic status more consistently, as this was largely absent across the reviewed studies despite evidence suggesting that socioeconomic context may influence opportunities for PA [110].

### 4.4. Limitations

There are several limitations that should be considered when interpreting the findings of this review. Notably, the small number of included studies limited the generalisability of findings and reflects the emerging nature of research in this area. The search strategy focused solely on terms reflecting the multidimensional nature of PL, which may have limited the number of studies retrieved. The studies included in this review were predominantly cross-sectional, which restricted the ability to infer causality. Additionally, heterogeneity in measurement tools and study methodologies made comparisons difficult and precluded meta-analysis. Finally, there is also potential for publication bias, as only peer-reviewed studies were included, and possible language bias, due to the restriction to English-language publications.

## 5. Conclusions

Globally, childhood obesity is increasing at an alarming rate [111]. Childhood is considered a critical stage of life for promoting and establishing healthy lifestyle behaviours [112], and PA levels positively track from early childhood into adolescence and adulthood [113]. In recent years, the concept of PL has gained increasing international political attention and has been integrated into several educational and sport policies [47,114]. However, the direct pathways linking PL to health outcomes requires further exploration. This paper aimed to address a gap by providing a systematic review of the current literature by examining the availability and effectiveness of PL assessment tools and evaluating the relationship between PL and the risk of obesity in children. Based on the evidence presented, significant gaps remain that warrant future research investigation.

It is clear from the literature review that there are many available tools. However, what is also evident is a lack of consistency in approaches between these tools in terms of their domain framework and how each domain is measured. This may lead to confusion for stakeholders when determining which tool is most appropriate for their needs and may hinder comparison between studies. While existing tools offer valuable insights, each tool presents limitations that should be considered when applied in practice. These findings should not be interpreted as a definitive judgement on current measures, but rather as an opportunity to support the development of a standardised and holistic approach to assessing PL. The research community should work towards establishing a common framework, such as the SOLO taxonomy or an alternative model, in order to promote greater coherence and comparability across PL assessment.

When considering the effectiveness of available tools, some contradictory findings may be made. These tools can be considered effective because they all provide a score for PL and this score can be tracked over time individually and in groups. However, scoring systems are not based on an agreed definition of PL; therefore comparisons between tools are impossible and perhaps of greater concern is that the tools may not be correctly or effectively measuring PL in children. Further modifications to assessment methods are warranted, and the research community plays an important role in this process. It could be argued that advances in wearable technologies, artificial intelligence and data analytics could provide an effective solution that overcomes the limitations of existing methods.

Perhaps of most concern is the lack of supporting evidence regarding the statistical relationship between PL and the risk of obesity in children. Despite frequent reporting, it remains unclear if increasing levels of PL will favour anthropometric measures in children. The findings of this review suggest that the hypothesised pathway linking PL, PA and adiposity remains largely theoretical and caution against inferring a direct relationship given the current evidence base. This paper also highlighted significant methodological differences in current studies and the under-reporting of certain anthropometric data. The implications of this are stark in terms of the value of existing PL interventions that are aimed at reducing the risk of childhood obesity. Further empirical research, particularly longitudinal and experimental studies, is essential to further inform stakeholders and to establish whether a direct pathway linking PL, PA and reduced adiposity exists. Future studies should also report contextual factors such as socioeconomic status, particularly when examining younger children, whose opportunities for PA are more strongly shaped by family and environmental influences.

It is imperative and urgent that researchers and practitioners address these significant challenges immediately and effectively. Failure to do so will further negatively impact the lives of young people worldwide as the global obesity epidemic continues to worsen at an alarming rate.

## Figures and Tables

**Table 1:** Summary of selected research studies investigating the availability and effectiveness of PL assessment tools and the relationship between PL and childhood obesity. References are presented in chronological order. Details included relate to the participants involved (F: female, M: male); age cohort examined; weight status (NW: normal weight, OW/O: overweight or obese); assessment tool adopted; anthropometric measure (BW: body weight; BMI: body mass index; %BF: percentage body fat; CRF: cardiorespiratory fitness; FM: fat mass; FFM: fat-free mass; %SMM: percentage skeletal muscle mass; WC: waist circumference); statistical test used; statistical variables (FMS: fundamental movement skill; MVPA: moderate-to-vigorous physical activity; PA: physical activity; PC: physical competence; K&U: knowledge and understanding); statistical result; and key conclusion. Unrep = unreported.

Ref	Year	Participants		Age	Weight Status		Assessment Tool	Obesity Indicator								Statistical Test	Statistical Variable(s)	Statistical Result	Key Conclusion
		M	F		NW	OW/O		BW	BMI	%BF	CRF	FM	FFM	%SMM	WC				
[83]	2017	78	67	9–12	unrep	unrep	PLAYbasic	•	•	•	•				•	Pearson’s correlation Linear regression	BMI and FMS (PLAYbasic)	(r −0.15 to −0.30; *p* < 0.05)	Health indicators in children are associated with FMSs.
[84]	2018	4160	4183	8–12	5307	3036	CAPL	•	•						•	Chi-squared goodness of fit	CAPL domain scores and BMI category	F(3, 4037) = 29.2, *p* < 0.001, F(3, 4077) = 25.1, *p* < 0.001,	The association between children’s age and PL is negligible.
[85]	2020	109	113	10.7 (±1.0)	unrep	unrep	PLAY tools	•	•	•	•		•			Linear regression Shapiro–Wilk test	BC and PL	*R*^2^ = 0.23	Higher PL was associated with favourable health indicators.
[86]	2021	63	72	8–12	83	52	CAPL-2	•	•			•	•			Kolmogorov–Smirnov and Levene independent *t*-test Mann–Whitney U-test Pearson’s and Spearman’s correlation	PL and BMI	r = −0.466, *p* < 0.012	Non-overweight children presented higher levels of PL than their overweight counterparts.
																	PL and FM	r = −0.478, *p* < 0.012	
																	PL and %FM	r = −0.491, *p* < 0.012	
																	PC and FM	r = −0.471, *p* < 0.012	
																	PC and %FM	r = −0.507, *p* < 0.012	
																	K&U and FM	r = −0.139	
																	K&U and %FM	r = −0.152	
[87]	2023	675	685	8–12	1085	206	CAPL-2	•	•		•				•	Independent *t*-test Chi-squared test MANOVA Spearman’s correlation Pearson’s correlation Multivariate logistic regression Partial Eta Squared	PL and BMI	r =−0.171 to −0.217,	Children with normal weight demonstrate a higher level of PL and domain score than those with overweight and obesity.
																	K&U and BMI	*p* < 0.01 r = 0.006	
[88]	2023	9	4	11.7 (±1.09)	0	13	CAPL-2	•	•	•	•			•		Shapiro–Wilk test Correlation matrices	PL and BMI PL and %BF	(−0.3 ± 0.3, *p* ≤ 0.01) (−3.8 ± 4.9, *p* ≤ 0.01) (+1.5 ± 1.7 mL·min·kg^−1^, *p* ≤ 0.01) (+4.6 ± 13.7 min/day)	Initiating multidimensional interventions to develop PL in overweight and obese adolescents may be a promising prospect to enable an increase in their MVPA and improve their long-term health.
[89]	2023	53	32	12.1 (±0.4)	72	11	F-PPLI	•	•		•	•		•		Shapiro–Wilk Mann–Whitney U test Linear regression Pearson’s correlation	PL and MVPA PL and %FM PL and %SMM PL and CRF	r = 0.38; *p* ≤ 0.01 r = 0.43; *p* ≤ 0.01 r = 0.36; *p* ≤ 0.01 r = −0.40; *p* ≤ 0.01	The level of PPL is positively associated with CRF, MVPA and %SMM and negatively associated with %FM.
[90]	2024	62	73	10.09 (±0.76)	78	58	CAPL-2	•	•		•					Regression modelling	PL and BMI	*p* = 0.018	Children of normal weight achieved higher level of PL than children with overweight or obesity.
[91]	2024	258	181	10–16	197	242	CAPL-2	•	•							Chi-squared test	Boys’ motivation and PA Motivation and BMI Knowledge and PA	(χ^2^ = 12.403, *p* < 0.006) (Effect size = 0.198) (χ^2^ = 60.460, *p* < 0.001)	Children with a healthier BMI tend to score higher in the motivation domain of PL.

**Table 2:** Summary of validity and reliability evidence (PLAY: Physical Literacy Assessment for Youth; CAPL: Canadian Assessment of Physical Literacy; PPLI: Perceived Physical Literacy Inventory). Unrep = unreported.

Ref	Assessment Tool	Type of Validity	Validity Result	Type of Reliability	Reliability Result
[73]	PPLI	Content	Items were presented generally allowing for cross-population use. AVE = 0.43 to 0.54	Internal consistency	CR > 0.60
		Convergent	AVE = 0.43 to 0.54	Item factor correlations	r = 0.48 to 0.66
[103]	PLAYfun	Construct	CFI = 0.95, RMSEA = 0.55	Inter-rater	ICC = 0.87
				Internal consistency	Item total correlations varied from 0.47 to 0.83
[104]	CAPL (PLKQ)	Content	Supports content validity; PLKQ increases with age and fitness knowledge. No significant correlation with gender or movement skill	Test–retest reliability	Reliability of the PLKQ total score was strong (r = 0.82 and 0.69 over the 2- and 7-day intervals). Adjusting the correlation by age did not alter the reliability (r = 0.60 and 0.70 over the 2- and 7-day intervals).
[105]	CAPL	Context	Original knowledge tool showed ceiling effects; revised PLK tool improved age relevance	unrep	unrep
		Construct	Supported by age-related progression in physical competence and knowledge domain		
[101]	PLAY	Construct	Correlation between age, PLAYbasic and PLAYfun (r = 0.16 to r = 0.32)	Inter-rater	PLAYfun and PLAYbasic show moderate-to-excellent inter-rater reliability (ICC = 0.87)
			Correlation between PLAYbasic and PLAYfun (r = 0.86, *p* < 0.001)	Internal consistency	Acceptable internal consistency; Cronbach’s alpha (α) and omega (ω) coefficients ranged from 0.80 to 0.87. Environmental and self-description subscales had the lowest α, while PLAYfun had the highest
		Convergent	Association between PLAYfun and PLAYparent (r = 0.41, *p* < 0.001)		
			Association between PLAYparent and PLAYself (r = 0.41, *p* < 0.001)		
[102]	PLAYself	Convergent	PLAYself has positive correlation with PLAYfun (r = 0.52, *p* < 0.001)	Internal consistency	Cronbach’s alpha = 0.80 for environmental and self-description components
				Test–retest reliability	Environmental subscale ICC = 0.81; self-description subscale ICC = 0.87

## Data Availability

Data will be made available on reasonable request to the corresponding author.

## Supplementary Materials

The following supporting information can be downloaded at: https://www.mdpi.com/article/10.3390/ijerph23050562/s1. Table S1: Joanna Briggs Institute (JBI) critical appraisal results for included and excluded studies. The table presents the methodological quality assessment scores for each study alongside reasons for exclusion where applicable; Table S2: PRISMA 2020 checklist [115].

## Abbreviations

The following abbreviations are used in this manuscript:

%BF	Percentage Body Fat
%SMM	Percentage Skeletal Muscle Mass
60 MKC	60 Minute Kids’ Club
BMI	Body Mass Index
BW	Body Weight
CAEPL	Chinese Assessment and Evaluation of Physical Literacy
CAMSA	Canadian Agility and Movement Skills Assessment
CAPL	Canadian Assessment of Physical Literacy
CAPL-2	Canadian Assessment of Physical Literacy Second Edition
CMOPL	Conceptual Model of Observed Physical Literacy
CRF	Cardiorespiratory Fitness
FFM	Fat-Free Mass
FM	Fat Mass
FMSs	Fundamental Movement Skills
IPLA	International Physical Literacy Association
K&U	Knowledge and Understanding
MVPA	Moderate-to-Vigorous Physical Activity
PA	Physical Activity
PACER	Progressive Aerobic Cardiovascular Endurance Run
PC	Physical Competence
PL	Physical Literacy
PLAY	Physical Literacy Assessment for Youth
PLKQ	Physical Literacy Knowledge Questionnaire
PLOT	Physical Literacy Observation Tool
PPLI	Perceived Physical Literacy Inventory
PRISMA	Preferred Reporting Items for Systematic Reviews and Meta-Analyses
SOLO	Structure of Observed Learning Outcomes
WC	Waist Circumference
WHO	World Health Organization
