# Supplementary material for: Physical Literacy and Obesity Risk in Children: A Systematic Review
Source: Int J Environ Res Public Health. 2026 Apr 27;23(5):562. doi: 10.3390/ijerph23050562 (PMC13206084; doi:10.3390/ijerph23050562)
Supplement: Supplementary file 1 [file ijerph-23-00562-s001.zip › Supplementary Table S1.pdf]

Supplementary Table S1. Joanna Briggs Institute (JBI) critical appraisal results for included and excluded studies. The table presents the methodological quality assessment scores for each study alongside reasons for exclusion where applicable.

**JBI for Cross-Sectional Studies**											
**Author, Year**	**Q1**	**Q2**	**Q3**	**Q4**	**Q5**	**Q6**	**Q7**	**Q8**	**Q9**	**Overall Appraisal**	**Reason for Exclusion**
Comeau et al., 2017	Yes	Yes	Yes	Yes	Unclear	Unclear	Yes	Yes	N/A	Include	N/A
Delisle Nyström et al., 2018	Unclear	Yes	Yes	Yes	Unclear	Unclear	Yes	Yes	N/A	Include	N/A
Caldwell et al., 2020	Yes	Yes	Yes	Yes	Unclear	Unclear	Yes	Yes	N/A	Include	N/A
Mendoza-Muñoz et al., 2021	Yes	Yes	Yes	Yes	Unclear	Unclear	Yes	Yes	N/A	Include	N/A
Liu et al., 2023	Yes	Yes	Yes	Yes	Unclear	Unclear	Yes	Yes	N/A	Include	N/A
Nezondet et al., 2023a	Yes	Yes	Yes	Yes	Unclear	Unclear	Yes	Yes	N/A	Include	N/A
Urbano-Mairena et al., 2024	Yes	Yes	Yes	Yes	Unclear	Unclear	Yes	Yes	N/A	Include	N/A
Muñoz-Urtubia et al., 2024	Yes	Yes	Yes	Yes	Unclear	Unclear	Yes	Yes	N/A	Include	N/A
Flanagan et al., 2018	Yes	Yes	No	No	No	No	Unclear	Yes	N/A	Exclude	Small sample size (n=28)<br>Does not measure PL directly
**JBI for Quasi-Experimental Studies**											
Mendoza-Muñoz et al., 2022	Yes	Unclear	No	Yes	Yes	Yes	Unclear	Yes	Yes	Exclude	Small sample size (n = 57)<br>Pilot study design<br>Short intervention (4 weeks)<br>Single school sample<br>No randomisation<br>No long-term follow-up<br>Not focused on obesity outcome
(Nezondet et al., 2023b)	Yes	Yes	Unclear	Unclear	Yes	Unclear	Yes	Yes	Yes	Include	N/A
